# Health literacy and changes in pattern of drug use among participants at the Stockholm Needle Exchange Program during the COVID-19 pandemic

**DOI:** 10.1186/s12954-021-00499-z

**Published:** 2021-05-10

**Authors:** K. Lindqvist, C. Wallmofeldt, E. Holmén, A. Hammarberg, M. Kåberg

**Affiliations:** 1 Stockholm Needle Exchange, Stockholm Centre for Dependency Disorders, Stockholm, Sweden; 2 Norra Stockholms Psykiatri (Psychiatry of Northern Stockholm), S:t Görans Hospital, Stockholm, Sweden; 3 Centre for Psychiatry Research, Department of Clinical Neuroscience, Karolinska Institutet, Stockholm, Sweden; 4 Stockholm Centre for Dependency Disorders, Stockholm Health Care Services, Stockholm County Council, Stockholm, Sweden; 5 Department of Medicine Huddinge, Division of Infection and Dermatology, Karolinska Institutet, Karolinska University Hospital Huddinge, Sprututbytet, S:t Görans sjukhus, Akutvägen 29, 112 81 Stockholm, Sweden

**Keywords:** COVID-19, SARS-CoV-2, Health literacy, People who inject drugs, Needle exchange program, Harm reduction

## Abstract

**Background and aims:**

People who inject drugs may be particularly vulnerable to the severe acute respiratory syndrome coronavirus 2 (SARS-CoV-2) due to underlying health problems, stigma and social vulnerabilities. Harm reduction services, including needle exchange programs (NEP), have been subjected to varying degrees of disruption in the world, especially in the beginning of the coronavirus disease 2019 (COVID-19) pandemic. Compared to responses in other countries, Sweden’s initial strategy toward limiting the spread and impact of COVID-19 was less restrictive to its citizens with no imposed general societal lockdown. In this study, we investigate changes in drug use patterns, utilization of NEP associated health services, COVID-19 health literacy and the prevalence of SARS-CoV-2 antibodies among NEP clients in Stockholm during the COVID-19 pandemic.

**Methods:**

NEP visits and services provided (needles/syringes, HIV and hepatitis C tests and treatment, naloxone distributed) and overall mortality among NEP clients between January 1 and October 31, 2020, were used for trend analyses in comparison with corresponding 2019 data. Between July 27 and October 2, 2020, NEP clients (*n* = 232) responded to a 27 item COVID-19 Health Literacy Questionnaire. SARS CoV-2 IgG antibody tests (*n* = 779) were performed between June 15 and October 31, 2020.

**Results:**

During the COVID-19 pandemic number of clients, client visits, naloxone distribution and HCV tests remained stable compared to 2019, while distribution of needles/syringes increased (*p* < 0.0001); number of HIV tests and HCV treatments decreased (*p* < 0.05); and mortality decreased (< 0.01). Overall, the level of health literacy concerning transmission routes and protective measures was high. SARS-CoV-2 antibody prevalence was 5.4% (95% CI 4.0–7.2).

**Conclusions:**

The Stockholm NEP managed to maintain a high level of clients and services during the pandemic. In general, COVID-19 health literacy was adequate and the overall SARS-CoV-2 antibody prevalence was low compared to the general population, which highlights a need for prioritized and targeted COVID-19 vaccination among PWID.

## Background

People who inject drugs (PWID) may be particularly vulnerable to the severe acute respiratory syndrome coronavirus 2 (SARS-CoV-2) due to underlying health problems, stigma, social marginalization and higher economic and social vulnerabilities, including lack of access to stable housing and health care [1–3]. Additionally, drug use often takes place in environments where individuals gather and both drugs and equipment for drug use may be shared, which in turn may increase the risk of acquiring and spreading SARS-CoV-2 [2].

The availability of harm reduction services, including needle exchange programs (NEP), has been subjected to varying degrees of disruption in the world, especially in the beginning of the coronavirus disease 2019 (COVID-19) pandemic. Some NEP sites have closed or reduced opening hours although other adjustments due to restrictions put in place in society at large (e.g., minimizing face-to-face contact, physical distancing mandates, travel restrictions, etc.) have been more commonly noted [4–6]. A study examining the effects of COVID-19 on availability of NEP in England (March–April 2020) showed that 91% out of 115 sites providing NEP services remained open, with reduced opening hours or other access restrictions [4]. Furthermore, a substantial decrease in clients, number of visits and numbers of needles distributed were noticed.

According to the European Monitoring Centre for Drugs and Drug Addiction (EMCDDA), some drug trafficking routes in Europe were disrupted in the early days of the pandemic with increased prices for some illegal drugs as a consequence [6]. EMCDDA also concludes that there were changes in drug use patterns due public health policies recommending or mandating social distancing and reduced social contact [7]. These changes urgently require monitoring of potentially negative effects among PWID.

Compared to the responses in most other countries, Sweden’s initial strategy toward limiting the spread and impact of COVID-19 has been less restrictive to its citizens [8]. The Public Health Agency of Sweden did not recommend or impose a general lockdown or the use of face masks in public areas. Instead, physical distancing was recommended but not mandatory. However, stricter regulations were put in place for public gatherings, events and for bars and restaurants [8, 9]. Sweden’s strategy to mitigate the spread of COVID-19 was considered unique compared to other countries, both in and outside of Europe, where considerable stricter physical distancing guidelines were enforced, including lockdowns and mandatory use of face masks in public areas [9].

In mid-August 2020, there were 83,852 registered cases (830 per 100,000) and a total of 5,776 deaths (57.1 per 100,000) in Sweden [9]. While the number of diagnosed cases per 100,000 during the first wave of COVID-19 peaked in June (partly explained by an overall increase in testing), the number of deaths per day peaked in mid-April to then gradually decrease. However, from mid-October cases in Sweden increased yet again, following the trend of the second wave of SARS-CoV-2, as seen overall in Europe. By the end of October, there were 132,029 registered cases and 6,001 deaths due to COVID-19 in Sweden [10].

Health literacy is defined as "the degree to which individuals have the capacity to obtain, process, and understand basic health information and services needed to make appropriate health decisions" [11]. Previous studies have noted that some PWID populations have low levels of health literacy and deficient knowledge on disease symptoms and management, which may result in challenges to take informed decisions on drug use, drug use-related risks and general health-seeking behavior [12–15]. However, it should also be acknowledged that health services may not be easily accessible or targeted to PWID’s specific needs, which in turn may limit healthcare utilization. NEP provides both an infrastructure and opportunities to impart evidence-based information in order to improve clients’ general health literacy, which could be applied to COVID-19.

In this study, we aim to gain more knowledge of the possible harms associated with drug use and COVID-19 among PWID in the Stockholm NEP. In order to better understand, customize and enhance preventive measures, we thus aim to examine COVID-19 health literacy, changes in drug use patterns, NEP access and utilization of NEP associated health services in relation to the COVID-19 pandemic and the COVID-19 imposed restrictions. Furthermore, we investigate the prevalence of SARS-CoV-2 antibodies among NEP clients over time as a marker for SARS CoV-2 exposure.

## Methods

The Stockholm NEP consist of two fixed sites in the center of Stockholm and one mobile unit. The NEP offer sterile injection equipment (i.e., needles/syringes and paraphernalia), testing for hepatitis A, hepatitis B (HBV), hepatitis C (HCV) and HIV. Furthermore, vaccination, risk reducing counselling and treatment for infectious diseases, including HCV and HIV treatment, referrals to social services and drug treatment services, including opioid agonist treatment (OAT), is provided. In 2018, a take-home naloxone (THN) program was implemented providing overdose prevention education and distribution of nasal naloxone. The NEP is staffed by physicians and nurses specialized in general practice, infectious diseases and psychiatry/substance use disorders, counsellors and midwives.

The Stockholm NEP account for almost half of all NEP visits in Sweden. In 2019, 1843 unique clients made a total of 24 305 visits at the Stockholm NEP. This represented 43% of all clients and 48% of all visits registered in the national quality register InfCare Needle Syringe Program that covered 97% of all NEP clients in Sweden in 2019. Clients are registered with their unique Swedish personal identity number. At admission, all respond to a 34-item questionnaire regarding sociodemographic data and injection risk behavior, described in detail in previous publications [16–18]. Questionnaires and tests for HIV and hepatitis are repeated at an interval of 3–6 months. At every visit, clients report the drug they used at last injection.

In this study, data on visits, services provided (i.e., distributed needles/syringes, HIV/hepatitis tests, HCV treatment, naloxone distributed) and overall mortality between January 1 and October 31, 2020, were used for trend analyses in comparison with corresponding data from 2019. A complementary face-to-face COVID-19 Health Literacy Questionnaire was introduced between July 27th and October 2nd with 27 questions regarding demographic data (level of education and housing conditions), health literacy on COVID-19 (transmission routes, risk factors for transmission and disease, knowledge on recommendations and personally applied protective measures and perceived exposure to COVID-19), change in drug use patterns, risk behaviors and contacts with health care. Questions were partly adapted from the EMCDDA European Web Survey on Drugs [7]. Data on mortality were automatically reported from the Swedish Board of Health and Welfare’s Death Registry to the national population register, which is linked to the Stockholm NEP medical charts.

### SARS CoV-2 IgG Ab test

SARS CoV-2 IgG antibody (Ab) tests were performed between June 15 (when first accessible within out-patient care in Stockholm) and October 31, 2020. Serological tests for SARS CoV-2 Ab test were performed routinely at the Karolinska University Hospital Laboratory.

### Statistics

All data from the NEP quality register were exported and analyzed using Microsoft Excel and JMP 15 (SAS Institute Inc., Cary, NC, USA). Descriptive characteristics are presented as frequencies and percentages. Chi-square or Fisher’s exact two-tailed test were used for categorical variables and the Wilcoxon rank-sum test for continuous values to test for differences in characteristics. A *p* value < 0.05 was considered as statistically significant.

## Results

### Access to NEP services during the covid-19 pandemic

Between January 1st and October 31st 2020, there were a total of 1,719 clients making 19,369 visits at the Stockholm NEP (Table 1).

**Table 1:** Service utilization at the Stockholm needle exchange program January–October, 2019/2020

	2019		2020		*p* value
	(*n*)	(*n*/client)	(*n*)	(*n*/client)	
NEP visits					
Clients	1,634	–	1,719	–	
Number of visits	18,936	11.6	19,369	11.3	0.44
Number of visits at other NEP	692	0.42	500	0.29	< 0.001
Distribution of needles/syringes					
Distributed syringes	395,656	242	601,969	350	< 0.0001
Distributed needles	452,919	277	718,051	418	< 0.0001
HIV/hepatitis					
HIV tests	2280	1.4	2145	1.2	< 0.05
HCV tests	600	0.4	585	0.3	0.26
HBV tests	672	0.4	546	0.3	0.0001
New HIV cases	3	0.002	2	0.001	0.68
New HCV cases	84	0.05	78	0.05	0.47
New HBV cases	1	0.0006	1	0.0006	1.0
Initiated HCV treatments	78	0.12^†^	54	0.08^†^	< 0.05
Take home naloxone (THN)					
New clients in THN program	226	0.16*	248	0.18*	0.35
Active clients in THN program	475	0.29	585	0.34	< 0.01
Naloxone doses distributed	1,686	3.5**	2,246	3.8**	0.26
Reported overdose reversals	329	0.69**	344	0.59**	0.10
Overdose with ambulance called	160	0.49°	166	0.48°	0.94
Overdose with rescue breathing	93	0.28°	123	0.36°	< 0.05
Mortality					
Deceased	41	0.03	19	0.01	< 0.01

^†^Based on a 40% viremic HCV prevalence

*Clients already in the THN program were excluded in this analysis

**Based on clients in THN program

°Based on number of reported overdoses

### Change in drug use patterns

**Fig. 1:**
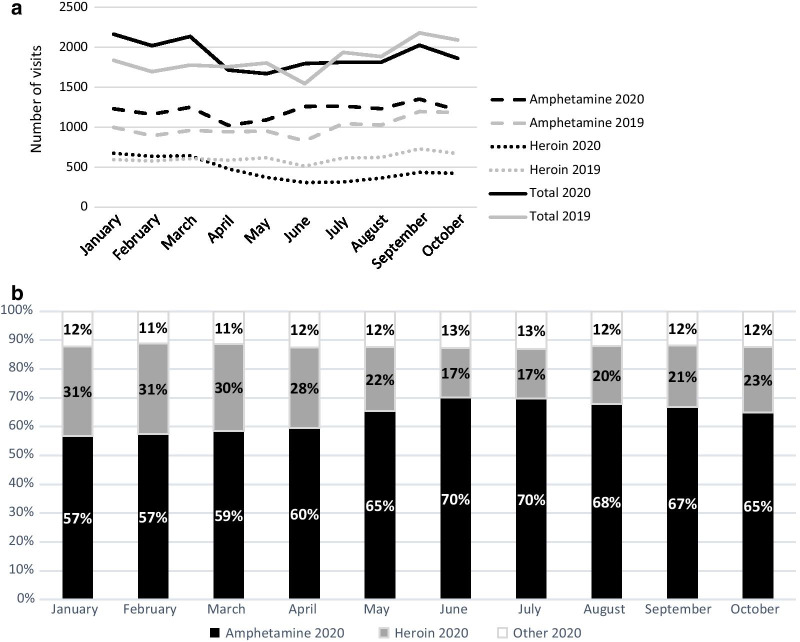
**a** Overall number of NEP visits/month January–October 2019/2020 and reported last injected drug at visits January–October 2019/2020. **b**. Proportions of reported last injected drug January–October 2020

There were no major changes in overall visits related to the number of clients between 2020 and 2019 (Table 1). However, the number of visits per month decreased by 18% between March and April 2020 (Fig. 1a). Specifically, a drop in NEP visits among clients reporting heroin as their last injected drug was noted in April 2020, in comparison with those injecting amphetamine (Fig. 1a). By June 2020, the proportion of heroin, as reported last drug at injection, had decreased by 45% (from 31 to 17%) mirrored by a 23% increase of amphetamine as last injected drug (Fig. 1b). Although Stockholm NEP clients still visited other NEP in different regions, there was a decrease in those visits in 2020 compared to 2019 (*p* < 0.001) (Table 1).

### Distribution of needles/syringes

Overall, a 50.9% (from 277 to 418) and 44.6% (from 242 to 350) increase in distribution of needles and syringes per person was noticed between January and October 2020 compared to the same period in 2019 (*p* < 0.0001). A sharp increase in mean monthly distribution of needles and syringes per person was noticed from February to April 2020, with a 49.3% (from 67 to 100) and 44.8% (from 58 to 84) increase, respectively (data not shown). This increased level of distribution was then maintained throughout the study period.

### Tests for HIV and hepatitis and treatment of HCV

Between January and October 2020, there was a decrease in HIV tests (*p* < 0.05) and HBV tests (*p* = 0.0001) compared to 2019, while the same level of HCV testing was maintained. No increased incidence of HIV, HBV or HCV was noted. However, there was a noticeable decrease in HCV treatment initiations (*p* < 0.05).

### Take home naloxone (THN), overdose response and deaths among NEP clients

There were no major disturbances seen regarding the THN program, with a continuous inclusion of new participants and distributed naloxone doses remaining at the same level as in 2019. Overall, the number of active clients in the THN program increased during 2020 (*p* < 0.01). There was no significant change to the number of reported overdose reversals between the two time periods. In terms of actions taken during overdose reversal, the frequency of an ambulance being called to the scene of the overdose, and overdose victims taken to the hospital remained constant, while rescue breathing was more frequently reported during the pandemic compared to the year before, 28.3% versus 35.8% (*p* < 0.05). The reported outcomes of THN interventions did not differ between 2020 and 2019, where 0.9% (3/344) and 0.6% (2/329) were reported as fatal overdoses, respectively (*p* = 1.0).

Between January and October 2020, the number of overall registered deaths among NEP clients were lower compared to the same period 2019, 1.1% versus 2.5% (*p* < 0.01) (Table 1).

### The COVID-19 health literacy questionnaire

Between July 27th and October 2nd, a total of 232 NEP clients responded to the Health Literacy Questionnaire (HLQ) study. Demographic data and HLQ responses are presented in Table 2. Overall, 177 (76.3%) of the participants were male. The mean age was 44.5 (IQR 33–55) years and 39.9 (IQR 31–49) years, for men and women, respectively. Almost a third (28.0%) of the participants were homeless (sleeping outdoors, in shelters or in public spaces), while 39.7% had a stable housing situation.

**Table 2:** Characteristics and answers from the COVID-19 Health Literacy Questionnaire, by main drug injected (*n* = 232)

	All	Main drug injected			*p* value Amphetamine/Heroin
		Amphetamine	Heroin	Other drug	
	*n* (%)	*n* (%)	*n* (%)	*n* (%)	
Total	232 (100)	144 (62.1)	65 (28.0)	23 (9.9)	
Gender (*n* = 232)					
Men	177 (76.3)	107 (74.3)	49 (75.4)	21 (91.3)	1.0
Women	55 (23.7)	37 (25.7)	16 (24.6)	2 (8.7))	
Age (*n* = 232)					
< 35	70 (30.2)	31 (21.5)	29 (44.6)	10 (43.5)	< 0.001
> 35	162 (69.8)	113 (78.5)	36 (55.4)	13 (56.5)	
Housing (*n* = 232)					
Stable	92 (39.7)	54 (37.5)	29 (44.6)	9 (39.1)	0.36
Irregular	75 (32.3)	50 (34.7)	17 (26.2)	8 (34.8)	0.26
Homeless	65 (28.0)	40 (27.8)	19 (29.2)	6 (26.1)	0.87
Education (*n* = 232)					
Not finished elementary (< 9 years)	29 (12.5)	17 (11.8)	9 (13.8)	3 (13.0)	0.66
Full elementary (9 years)	84 (36.2)	52 (36.1)	20 (30.8)	12 (52.2)	0.53
Upper secondary	92 (39.7)	61 (42.4)	25 (38.5)	6 (26.1)	0.65
University	27 (11.6)	14 (9.7)	11 (16.9)	2 (8.7))	0.17
Perceived COVID-19 transmission routes (*n* = 232)					
Injection paraphernalia	114 (49.1)	67 (46.5)	41 (63.1)	6 (26.1)	< 0.05
Inhalation paraphernalia	170 (73.3)	106 (73.6)	54 (83.1)	10 (43.5)	0.16
Drug solution	100 (43.1)	54 (37.5)	39 (60.0)	7 (30.4)	< 0.01
Cigarette	167 (72.0)	104 (72.2)	52 (80.0)	11 (47.8)	0.30
Through contact surfaces	190 (81.9)	119 (82.6)	58 (89.2)	13 (56.5)	0.30
Through droplet	215 (92.7)	134 (93.1)	64 (98.5)	17 (73.9)	0.18
Living together	170 (73.3)	107 (74.3)	51 (78.5)	12 (52.2)	0.60
Perceived effective protective measures (*n* = 232)					
Social distance	175 (75.4)	107 (74.3)	52 (80.0)	16 (69.6)	0.48
Hygiene routines	203 (87.5)	125 (86.8)	57 (87.7)	21 (91.3)	1.0
Face mask	118 (50.9)	75 (52.1)	34 (52.3)	9 (39.1)	1.0
COVID-19 vaccination	103 (44.4)	67 (46.5)	30 (46.2)	6 (26.1)	1.0
Treatment for COVID-19	116 (50.0)	76 (52.8)	35 (53.8)	5 (21.7)	1.0
Illicit drugs	71 (30.6)	54 (37.5)	15 (23.1)	2 (8.7)	0.06
Other	19 (8.2)	13 (9.0)	4 (6.2)	2 (8.7)	0.80
Knowledge of medical risk factors associated with COVID-19 infection (*n* = 217)					
Yes	142 (65.4)	87 (64.4)	38 (62.3)	16 (80.0)	0.87
No	75 (34.6)	48 (35.6)	23 (37.7)	4 (20.0)	
Reported personal risk factors (*n* = 232)					
None	33 (14.2)	19 (13.2)	8 (12.3)	6 (26.1)	1.0
COPD	11 (4.7)	9 (6.3)	2 (3.1)	0	0.51
Age	0	0	0	0	–
High blood presure	37 (15.9)	29 (20.1)	6 (9.2)	2 (8.7)	0.07
Diabetes mellitus	10 (4.3)	7 (4.9)	1 (1.5)	2 (8.7)	0.44
Obesity	18 (7.8)	12 (8.3)	4 (6.2)	2 (8.7)	0.78
Smoking	180 (77.6)	111 (77.1)	55 (84.6)	14 (60.9)	0.20
Immunodeficiency	12 (5.2)	8 (5.6)	1 (1.5)	3 (13.0)	0.28
Fear of contracting COVID-19 (*n* = 231)					
Yes	57 (24.7)	36 (25.2)	14 (21.5)	7 (30.4)	0.60
No	174 (75.3)	107 (74.8)	51 (78.5)	16 (69.6)	
Self-reported COVID-19 infection (*n* = 231)					
Yes, confirmed	7 (3.0)	7 (4.9)	0	0	0.10
Yes, suspected	58 (25.1)	39 (27.3)	15 (23.1)	4 (17.4)	0.61
No	132 (57.1)	75 (52.4)	44 (61.7)	13 (56.5)	< 0.05
Don’t know	34 (14.7)	22 (15.4)	6 (9.2)	6 (26.1)	0.28
Behavioral change if confirmed/suspected COVID-19 (*n* = 65)					
Social distance	27 (41.5)	20 (43.5)	5 (33.3)	2 (50.0)	0.56
Home isolation	25 (38.5)	20 (43.5)	5 (33.3)	0	0.56
Admitted to hospital	3 (4.6)	3 (6.5)	0	0	0.57
No change	25 (38.5)	17 (37.0)	7 (46.7)	1 (25.0)	0.55
SARS CoV-2 Ab (*n* = 227)					
Positive	15 (6.6)	14 (9.9)	1 (1.6)	0	< 0.05
Negative	207 (89.2)	123 (87.2)	63 (98.4)	21 (95.5)	
N/A	5 (2.2)	4 (2.8)	0 (0)	1 (4.5)	–
Change of injected drug (*n* = 231)					
Yes	30 (13.0)	12 (8.4)	13 (20.0)	18 (78.3)	< 0.05
No	201 (87.0)	131 (91.6)	52 (80.0)	5 (21.7)	
Aquired drugs through alternative sources (*n* = 229)					
No change	174 (76.0)	118 (83.7)	39 (60.0)	17 (73.9)	< 0.001
Used other suppliers	29 (12.7)	8 (5.7)	19 (29.2)	2 (8.7)	< 0.0001
Stopped using illicit drugs	11 (4.8)	4 (2.8)	7 (10.8)	0	< 0.05
Change in NEP visit frequency (*n* = 223)					
No change	149 (66.8)	90 (63.4)	42 (70.0)	17 (81.0)	0.42
More frequent	8 (3.6)	5 (3.5)	2 (3.3)	1 (4.8)	1.0
Less frequent	66 (29.6)	47 (33.1)	16 (26.7)	3 (14.3)	0.41
Increased sharing of needle/syringe (*n* = 232)					
Yes	11 (4.7)	6 (4.2)	4 (6.2)	1 (4.3)	0.51
No	221 (95.3)	138 (95.8)	61 (93.8)	22 (95.7)	
Shared needle/syringe during the past month (*n* = 231)					
Yes	34 (14.7)	24 (16.7)	8 (12.3)	3 (13.3)	0.53
No	197 (85.3)	120 (83.3)	57 (87.7)	20 (86.7)	
Increased sharing of paraphernalia (*n* = 229)					
Yes	14 (6.1)	10 (7.0)	4 (6.2)	0	1.0
No	215 (93.9)	133 (93.0)	61 (93.8)	21 (100)	
Shared paraphernalia during the past month (*n* = 229)					
Yes	64 (27.9)	44 (30.8)	17 (26.2)	5 (21.7)	0.62
No	165 (72.1)	99 (69.2)	48 (73.8)	18 (78.3)	
Healthcare avoidance (*n* = 232)					
Yes	34 (14.7)	17 (11.8)	11 (16.9)	6 (26.1)	0.38
No	198 (85.3)	127 (88.2)	54 (83.1)	17 (73.9)	
Had enough needles/syringes and other paraphernalia (*n* = 231)					
Yes	197 (85.3)	123 (85.4)	56 (87.5)	18 (78.3)	0.83
No	34 (14.7)	21 (14.6)	8 (12.5)	5 (21.7)	
Aquired needles/syringes through alternative sources (*n* = 232)					
Yes	45 (19.4)	27 (18.7)	12 (18.5)	6 (26.1)	1.0
No	187 (80.6)	117 (81.3)	53 (81.5)	17 (73.9)	
Experienced/heard that number of overdoses increased (*n* = 229)					
Yes	42 (18.3)	25 (17.6)	15 (23.1)	2 (9.1)	0.35
No	161 (70.3)	103 (72.5)	39 (60.0)	19 (86.4)	0.08
Don’t know	26 (11.3)	14 (9.9)	11 (16.9)	1 (4.5)	0.17
OAT (*n* = 232)					
Yes	54 (23.3)	19 (13.2)	28 (43.1)	7 (30.4)	< 0.0001
No	178 (76.7)	125 (86.8)	37 (56.9)	16 (69.6)	
Modified OAT provision (*n* = 54)					
Yes	35 (64.8)	15/19 (78.9)	14/28 (50.0)	6/7 (85.7)	0.07
No	19 (35.2)	4/19 (21.1)	14/28 (50.0)	1/7 (14.3)	

### Patterns of NEP visits and drug use

The majority (62.1%) of the HLQ participants reported amphetamine as their main injected drug, while 28.0% injected heroin. The remaining 9.9% injected other drugs, including, e.g., buprenorphine, methylphenidate and cocaine.

Overall, 66.8% reported no change in NEP visit patterns. Among those who reported any change (*n* = 74), 89.2% came less frequent while 10.8% increased their visit frequency.

**Fig. 2:**
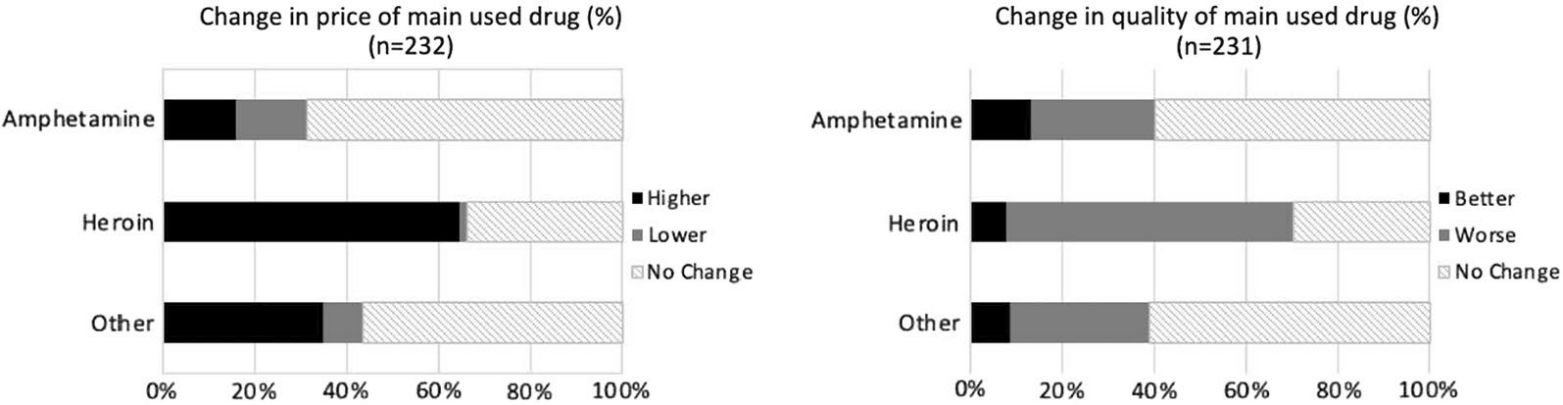
Reported change in price and quality of injected main drug among participants. A greater proportion of participants using heroin, compared to those using amphetamine, reported an increase in price (*p* < 0.0001) and worse quality of drugs (*p* < 0.0001)

Only 13.0% reported that they changed their main injected drug during the COVID-19 pandemic. Among participants primarily using heroin, 20.0% changed their main drug compared to 8.4% of those injection amphetamine (*p* < 0.05). The main reported reasons for shifting from heroin (mainly to amphetamine) were due to difficulties in acquiring heroin or increased price of heroin. Changes in price and perceived quality of drugs are presented in Fig. 2. Two thirds (64.6%) of participants using heroin reported a price increase and 62.5% reported quality deterioration of heroin during the COVID-19 pandemic (Fig. 2). Those using heroin were more subjected to changes in price and quality, compared to those using amphetamine (*p* < 0.0001).

Participants injecting heroin also reported changes in strategies to acquire drugs (*p* < 0.001), used other suppliers (*p* < 0.0001) or stopped acquiring illicit drugs altogether (*p* < 0.05) to a greater extent than participants using amphetamines.

### COVID-19 transmission and protective measures

All HLQ participants reported one or several perceived routes of transmission for COVID-19. The majority reported that COVID-19 was transmitted through droplets (92.7%); contact surfaces (81.9%) and living in close contact with someone (73.3%) (Table 2). Other perceived routes were through sharing of inhalation paraphernalia (73.3%); cigarettes (72.0%); needles/syringes and other paraphernalia (49.1%) or drug solution (43.1%). A higher proportion of participants using heroin believed that injection paraphernalia (*p* < 0.05) and drug solution (*p* < 0.01) were a route for COVID-19 transmission.

Correct hygiene practices including washing hands (87.5%); social distancing (75.4%); and using a face mask (50.9%) were the most frequent reported known protective measures for COVID-19. Almost half (44.4%) reported confidence in an effective future vaccine. Among a third (30.6%) of participants, there was also a perceived idea that some illicit drugs had a protective effect on COVID-19, with amphetamine most frequently mentioned, 71.8% (51/71).

Overall, 65.4% of participants reported knowledge on perceived risks associated with COVID-19. Risks mentioned were high blood pressure, cardiovascular disease, diabetes mellitus, chronic obstructive pulmonary disease (COPD), severe liver disease, being immunocompromised, obesity, old age and smoking tobacco. In terms of own risk factors, participants reported smoking tobacco (77.6%); high blood pressure (15.9%); obesity (7.8%); being immunocompromised (5.2%); COPD (4.7%) and diabetes mellitus (4.3%), while 14.2% reported no risk factor. Three out of four, (75.3%) stated that they were not worried about a possible exposure to or contracting COVID-19.

Level of education was not generally associated with level of health literacy. However, 79.4% (158/199) of participants with full elementary or higher education reported social distancing as an important measure, compared to 58.6% (17/29) of those without full elementary (*p* < 0.05). Furthermore, 41.4% (12/29) of those without full elementary education reported that illicit drugs had a protective effect on COVID-19, compared to 14.8% (4/27) among those with university studies (*p* < 0.05) (data not shown).

### Injection risk behaviors

Among the 232 participants in the HLQ, 85.3% reported that they had sufficient access to needles/syringes and other drug paraphernalia during the COVID-19 pandemic, while 14.7% reported insufficient access. A fifth, 19.4% (45/232), accessed needles and syringes from a different source than normally, 71.1% (32/45) from a peer, 17.8% (8/45) ordered over the internet and 11.1% (5/45) through a non-governmental organization (NGO). Accessing needles/syringes from a different source was associated with less frequent visiting patterns to the NEP (*p* < 0.05) (data nor shown).

Overall, 4.7% and 6.1% stated that they had shared needles/syringe and paraphernalia, respectively, more often during the COVID-19 pandemic. However, 85.3% and 72.1% had not shared needles/syringe or paraphernalia the past month. Less frequent visits were not associated with increased sharing of needles/syringes or other paraphernalia (data not shown).

### Contacts with other healthcare

Among participants receiving OAT, 64.8% (35/54) stated that OAT provision was somewhat modified during the pandemic, with OAT clinics offering temporary outdoor dispensing of medicine and less frequent visits for directly observed treatment, where 37.1% (13/35) reported increased provision of take-home doses.

In relation to the COVID-19 pandemic, 14.7% (34/232) stated that they avoided to seek general medical care mainly due to non-acute problems or less access to out-patient appointments 61.8% (24/34); mild COVID-19 symptoms 14.7% (5/34) and fear of being exposed to COVID-19 14.7% (5/34).

### SARS CoV-2 and perceived infection

The vast majority of participants in the HQL (227/232), were tested for SARS CoV-2 Ab. In the questionnaire, 3.0% (7/231) reported a confirmed and 25.1% (58/231) a suspected COVID-19 infection. However, only 6.6% (15/227) were SARS CoV-2 Ab positive. Among those with a positive SARS CoV-2 Ab test and a self-reported COVID-19 status (*n* = 13), 30.8% (4/13) reported a confirmed diagnosis, 30.8% (4/13) reported a suspected infection and 38.5% (5/13) no infection. Conversely, 93.1% (54/58) of those who suspected that they at some point were infected with COVID-19 did not have a positive SARS CoV-2 Ab test.

Of the 65 participants who reported a confirmed or suspected COVID-19 infection, 41.5% and 38.5% practiced social distancing and/or home isolation due to symptoms of illness. On the other hand, 38.5% reported that they did not change their behavior at all during illness.

The prevalence of SARS CoV-2 Ab was higher among participants injecting amphetamine compared to those injecting heroin, 9.9% versus 1.6% (*p* < 0.05). Housing situations was not associated with seroconversion (data not shown).

### Overall prevalence of SARS CoV-2 Ab, June to October

A total of 718 NEP clients were tested for SARS CoV-2 Ab between June 15 and October 31, 2020 (Table 3). Among the tested, 53.2% (382/718) injected mainly amphetamine and 27.7% (199/718) mainly heroin. Overall, there were 779 tests (55 clients were tested 2 times and 3 clients were tested 3 times) with an accumulated SARS CoV-2 Ab prevalence of 5.4% (95% CI 4.0–7.2). All clients tested multiple times were continuously SARS CoV-2 Ab negative.

**Table 3:** SARS CoV-2 antibody prevalence, June–October 2020, with 95% confidence intervals (CI)

Month	*n*	Positive (*n*)	Negative (*n*)	N/A (*n*)	Positive (%)	CI 95%
June	120	5	114	1	4.2	1.8–9.4
July	176	12	162	2	6.8	3.9–11.5
August	187	8	177	2	4.3	2.2–8.2
September	218	10	205	3	4.6	2.5–8.2
October	78	7	69	2	9.0	4.4–17.4
Accumulated	779	42	727	10	5.4	4.0–7.2

Amphetamine use was more prevalent among those who were SARS CoV-2 Ab positive, 61.9.% (26/42). The prevalence of SARS CoV-2 Ab was 6.2% (95% CI, 4.2–8.9) and 4.7% (95% CI, 2.5–8.3) among participants injecting amphetamine and those injecting heroin, respectively (*p* = 0.09).

## Discussion

In this study, we aimed to gain knowledge of COVID-19 health literacy and possible changes in drug use pattern and associated harms during the COVID-19 pandemic, in order to better customize and enhance preventive measures among PWID.

One of the overarching key components in harm reduction services and NEP addressing PWID, is continuous HIV/hepatitis surveillance through recurrent screening. While the Stockholm NEP’s operational hours remained unchanged despite COVID-19, with no overall decrease in client numbers or visits, testing for both HIV and HBV as well as initiation of HCV treatment decreased during the pandemic. A decrease in HIV and hepatitis testing was also noted in a study from the USA, investigating operational changes due to COVID-19 in 65 different NEP. A majority (84.6%) of the NEP remained open although 72.3% were operating under restricted hours which resulted in challenges in accessing safe injection equipment for PWID [19]. Additionally, only 26.1% continued offering testing for HIV or hepatitis. Also, a study examining the COVID-19 effects on NEP in England showed that the number of clients and number of visits decreased by 36% and the number of needles distributed decreased by 29% [4]. Furthermore, a study from Spain reported a 22% decrease in service users across all studied harm reduction centres and a 40% decrease in average needle distribution as well as a reduction in in HIV/hepatitis testing [4, 20].

Although our study examines a longer period of the COVID-19 pandemic, a decrease in number of needles/syringes distributed was not observed as seen in, e.g., the USA, England and Spain. On the contrary, there was an increase in the number of injection equipment distributed, reaching over 350 needles/syringes per person during the first ten months of 2020. These numbers are in line with the WHO strategy for HCV elimination, where > 300 needle/syringes distributed per PWID per year is proposed for an effective prevention of HIV and hepatitis transmission [21, 22]. A strategic increase in distributed needles/syringes in Stockholm may have facilitated the use of a peer-based “secondary needle exchange” where needles/syringes are disseminated to individuals that for different reasons neither want nor have the possibility to access the NEP themselves [23]. This hypothesis is strengthened by our data, showing that the majority of those who accessed needles/syringes from a source other than the NEP received it from a peer.

A combination of limited access to sterile injection equipment and decreased HIV/hepatitis testing, could lead to an increased risk of transmission of HIV and hepatitis. Efforts to maintain high access to harm reduction interventions, screening for infectious diseases and HIV/hepatitis treatment among PWID thus need to be reinforced rather than reduced in its capacities during a pandemic. However, the public health considerations between limiting the spread of COVID-19 (social distancing and lockdowns) on one hand and limiting transmission of viral hepatitis and HIV on the other may be conflicting and warrant further evaluation during the pandemic.

From the Health Literacy Questionnaire, we could conclude that the overall COVID-19 health literacy was high with the vast majority having correct knowledge about transmission routes, protective measures and personal risk factors. However, this knowledge did not necessarily reflect a willingness or ability to fully comply with the COVID-19 recommendations, with, e.g., over a third of the self-reported exposed participants reporting that they did not practice social distancing or home isolation. These difficulties might be explained by factors such as social marginalization, poor living conditions, psychiatric comorbidities [13] or some participants’ belief that they were less likely to contract COVID-19 or become severely ill if they did. These combined challenges require actions from both healthcare services and social services to provide PWID with better understanding and support to facilitate compliance to COVID-19 recommendation.

Interestingly, a third of the participants in the Health Literacy Questionnaire, believed that illicit drugs, in particular amphetamine, had a protective effect on COVID-19. Myths like this may introduce an increased risk for COVID-19 exposure if preventive measures are neglected. As a possible effect, amphetamine users at the Stockholm NEP were more exposed to COVID-19 (with a higher prevalence of SARS-CoV-2 Ab) compared to heroin users, although further studies are needed to strengthen causality. Myths, about drugs as a protective measure, like amphetamine in this study, is however not unique for the PWID population. A World Health Organization fact sheet specifically addressed and dispelled myths about “consuming alcohol destroys the virus that causes COVID-19”, “drinking strong alcohol kills the virus in the inhaled air” and “alcohol stimulates immunity and resistance to the virus” [24].

In our data, level of education was not an overall significant factor for COVID-19 related health literacy, although knowledge on social distancing was lower among those without full elementary education. This is in contrast to what we have found in previous studies, where an overall lower level of education was associated with, e.g., higher risk for sharing paraphernalia and acquiring HCV reinfection [17, 18]. These findings might be explained by the massive information efforts made on COVID-19 in society at large.

Changes in the illicit drug market, disruptions in harm reduction services with reduced naloxone provision may lead to higher risks of overdose deaths [25, 26]. Social distancing has also been pinpointed as a possible risk for fatal overdose, if opioids are used in settings without by-standers that may intervene in the event of an overdose [27, 28]. However, the COVID-19 pandemic’s impact on the THN program at the Stockholm NEP appears to be minor. Distribution of naloxone during the pandemic was higher than the same period in 2019, partly explained by an accumulated increased number of THN participants in 2020, as new participants were recruited at the same rate as before the pandemic. Other THN providers have reported changes to their training methods, such as using digital rather than in-person methods or replacing group trainings with one-to-one sessions [25]. The Stockholm NEP made few major changes to its usual training delivery, albeit the practical element of cardiopulmonary resuscitation (CPR) training was put on hold in order to follow recommendations to reduce close contact between healthcare personnel and clients. Still, the Stockholm NEP prioritized increased access to the THN program despite the challenges of providing training during the pandemic.

Unlike reports from emergency medical services in the USA, THN participants in the Stockholm NEP did not seem to be reluctant to administer naloxone nasally due to a perceived risk of COVID-19 infection [29]. Another study from the USA also noted that people who had overdosed were more likely to refuse transport to hospitals, possibly out of fear of exposure to COVID-19 [30]. This was however not observed in our study. Surprisingly, our data show that THN participants giving rescue breathing in an overdose situation increased during the pandemic, which could result in heightened risk for COVID-19 exposure. The underlying causes for this increase need to be investigated further and highlights the need for targeted COVID-19 information during THN training in combination with expanded protective measures, e.g., CPR pocket masks or face shields.

By the end of March, the Swedish Board of Health and Welfare recommended adjustments for OAT care related to the COVID-19 pandemic [31]. Factors to consider in order to reduce the spread of infection were to increase hygiene measures at delivery points and introduce fewer appointments and longer pickup intervals for medication, when medically justified, to avoid gathering of patients. These measures reflected what NEP clients themselves reported as modified OAT provision. On a parallel level, OAT clinics in Stockholm reported an overall increase of referrals between March and August, and clients in care from June to October (personal communication M. Sandell and N. Eriksson).

There have been signals of increased overdose deaths following the COVID-19 pandemic, and studies have emphasized scaling up overdose prevention programs in order to prevent fatalities [27, 32]. A study from the USA noted that overdose-related cardiac arrests rose sharply during April 2020 and remained elevated during the first months of the pandemic [33]. Somewhat surprisingly, we noticed a substantial decrease in number of deaths among Stockholm NEP clients during the pandemic. However, since there is a possible delay in mortality data during the pandemic period and no data on actual cause of death available through the NEP registry, it is hard to draw any firm conclusions of overall mortality. Nevertheless, the increased access to OAT in combination with the decreased quality of heroin, less use of heroin, transition from heroin use to amphetamine use and increased access to THN might possibly explain a decrease in drug related deaths [34]. These factors might also be a plausible explanation for the substantial decrease in visits of clients using heroin at the NEP. Adding to this, findings in Europe indicate an overall decline in drug use, localized shortages of heroin and an increase in replacement substances (with a noticeable increase in amphetamine use in Nordic cities), as well as an increase in attempts to access OAT as an effect of COVID-19 [7]. Despite this, our data showed that the number of reported overdose reversals remained on the same level in 2020, which is in contrast to the decreased use of heroin, and thus needs to be studied further to be fully understood.

In a recent report, The Swedish Public Health Agency assessed that the need for drug consumption rooms (DCR), in a Swedish context, should be investigated [35]. Data show that DCR reduce overdose risks, improve hygiene conditions and could be a setting for further educative efforts and other health related benefits, which all could be applied to the on-going COVID-19 pandemic [36–39].

Compared to the general population in Stockholm, SARS CoV-2 Ab prevalence was low among clients at the Stockholm NEP. During the study period, a total of 54.5% (718/1318) of the clients were tested, with an accumulated prevalence of 5.4% (95% CI 4.0–7.2). The prevalence in June (*n* = 120) was 4.2% (95% CI 1.8–9.4). In comparison, sampling of healthy blood donors in Stockholm in June (week commencing June 8th) showed a prevalence of SARS CoV-2 Ab of 11.4% (95% CI 6.6–17.8) [40]. Sampling in outpatient care in Stockholm the same week, based on residual material from blood samples taken on a medical indication other than COVID-19, showed a prevalence of 11.5% (95% CI 8.0–15.7) [41]. Furthermore, the prevalence of SARS CoV-2 Ab in Rinkeby-Kista, a comparatively low socioeconomic region in Stockholm with a higher than usual amount of people born outside of Sweden, the prevalence (*n* = 530) was 18.7% (95% CI 14.8–23.3) in June (week commencing June 22nd) [42]. Overall, PWID and the homeless population in the Stockholm NEP seem to have been less affected by COVID-19 compared to the general population in Stockholm. This is in contradiction to the supposed increased risk environments homeless PWID are exposed to, i.e., living on the streets, in shelters, often with unhygienic living conditions and injection drug use practices as a consequence. On the other hand, avoiding public transports during rush hours, avoiding regular close contacts (with e.g., known increased COVID-19 transmission rates at workplaces and within households) in combination with spending a considerable amount of time outdoors may have been protective factors.

It is not fully established to what extent mild infection or exposure to COVID-19 result in SARS CoV-2 seroconversion [43]. An Icelandic study noted that 91% of all that recovered from a SARS CoV-2 infection were seropositive, while a Swedish study discuss that a robust memory T cell response, even without detection of SARS CoV-2 Ab, may contribute to protection against severe COVID-19 infection [44, 45]. Thus, those who self-reported milder infection or exposure to COVID-19 in our study but without SARS CoV-2 seroconversion might still have been infected. Furthermore, the longevity of the antibody response is still unknown [43].

NEP constitute ideal settings to provide health interventions to PWID who generally access regular health care to a lesser extent than the population at large. Administration of other vaccines within NEP and harm reduction services has been highly successful, and taken into account the vulnerabilities in this population (e.g., co-morbidities, social instability and homelessness), PWID should be considered for prioritized early COVID-19 vaccination [46–48]. This may be of extra importance in settings where PWID initially have been less exposed to COVID-19, and thus are more susceptible in a preceding pandemic.

### Strengths, limitations and way forward

A strength of this study was that all NEP participants in Stockholm could be included through the Stockholm NEP database which provided solid data on NEP attendance and service provision before and during the pandemic. To our knowledge, specifically targeting a large cohort of PWID for SARS CoV-2 Ab testing is unique. However, there are several limitations. Since data are regional and Sweden’s COVID-19 policies during the first wave of COVID-19 differed substantially from other countries, our conclusions may not be representative and inferable to other NEP/PWID populations. Furthermore, the Health Literacy Questionnaire was based on self-reported data which may introduce a risk for underreporting, recall- or social desirability response bias. Another limitation is the lack of, to date, published reports on COVID-19 health literacy among the general Swedish population for comparison. Still, we conclude that health literacy among PWID was high in this study given the high level of correct answers. Lastly, the long-term effects of the COVID-19 pandemic on PWID cannot yet be fully assessed, especially taking into account recurrent waves of COVID-19. It is thus of the utmost importance to continue to monitor COVID-19 related changes in drug-use pattern, changes in risk behaviors and the possible negative effects on PWID’s health. Further studies are needed to ascertain whether the findings of this study could be applicable to PWID in general when looking at e.g., SARS-CoV-2 seroconversion rates, effect of COVID-19 vaccination and overall measures taken in order to minimize risk behavior and the spread of PWID related infectious diseases.

## Conclusion

In conclusion, the Stockholm NEP, which accommodate almost half of all NEP participants in Sweden, managed to maintain a high level of clients and client visits during the pandemic. Furthermore, there was a high and continuous provision of services including increased numbers of distributed needles/syringes and take-home naloxone although a decrease in HIV and hepatitis testing was noticed. In general, COVID-19 health literacy concerning transmission routes and protective measures was high among participants. The overall SARS-CoV-2 antibody prevalence among NEP clients was low compared to the general population, which highlights a need for prioritized and targeted COVID-19 vaccination among PWID.

## Data Availability

The datasets used by the study are available from the corresponding author on reasonable request.

## Abbreviations

Ab: Antibody
CI: Confidence interval
COPD: Chronic obstructive pulmonary disease
COVID-19: Coronavirus disease 2019
CPR: Cardiopulmonary resuscitation
DCR: Drug consumption room
EMCDDA: European Monitoring Centre for Drugs and Drug Addiction
HIV: Human immunodeficiency virus
HBV: Hepatitis B
HCV: Hepatitis C
HLQ: Health Literacy Questionnaire
IQR: Interquartile range
NEP: Needle exchange programs
NGO: Non-governmental organization
OAT: Opioid agonist treatment
PWID: People who inject drugs
SARS-CoV-2: Severe acute respiratory syndrome coronavirus 2
THN: Take-home naloxone
US: United States
